# Supplementary material for: A Workflow for Protein Structure Determination From Thin Crystal Lamella by Micro-Electron Diffraction
Source: Front Mol Biosci. 2020 Aug 4;7:179. doi: 10.3389/fmolb.2020.00179 (PMC7417479; doi:10.3389/fmolb.2020.00179)

Supplementary Information

for

A Workflow for Protein Structure Determination from Thin Crystal Lamella by Micro-Electron Diffraction

Emma V. Beale[1†], David G. Waterman[2,3†*], Corey Hecksel[4†], Jason van Rooyen[4], James B. Gilchrist[4], James M. Parkhurst[1], Felix de Haas[5], Bart Buijsse[5], Gwyndaf Evans[1*], Peijun Zhang[4,6,7*]

This document includes

Supplementary figures 1-3
Supplementary Table 1

Continuous rotating electron diffraction data collection script (Felix de Haas, Thermo Fisher Scientific)

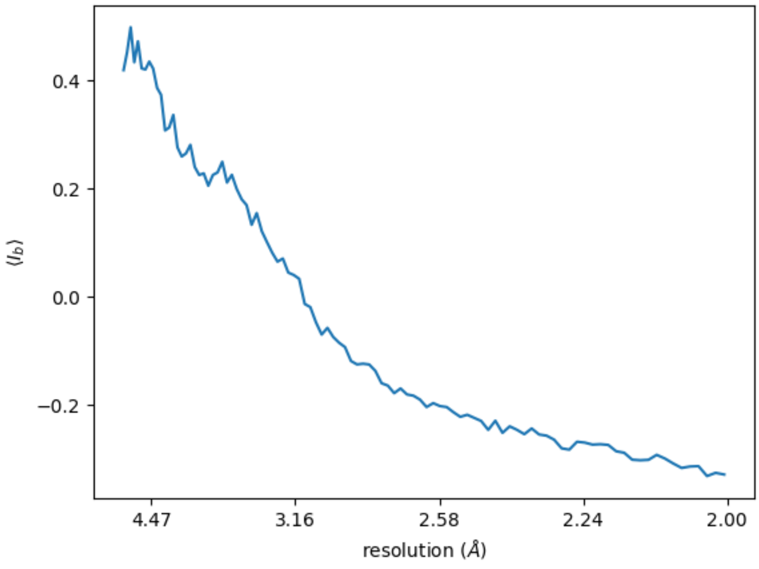

Supplementary Figure 1 | The average background values from a single frame of a representative nanocrystal dataset. The average background values were calculated in resolution shells by the program *dials.background* for image 18 of one nanocrystal dataset collected with a 20 µm condenser aperture. This diffraction pattern exhibits the strongest negative bias for that dataset, with average background levels below zero at resolutions beyond about 3.1 Å.

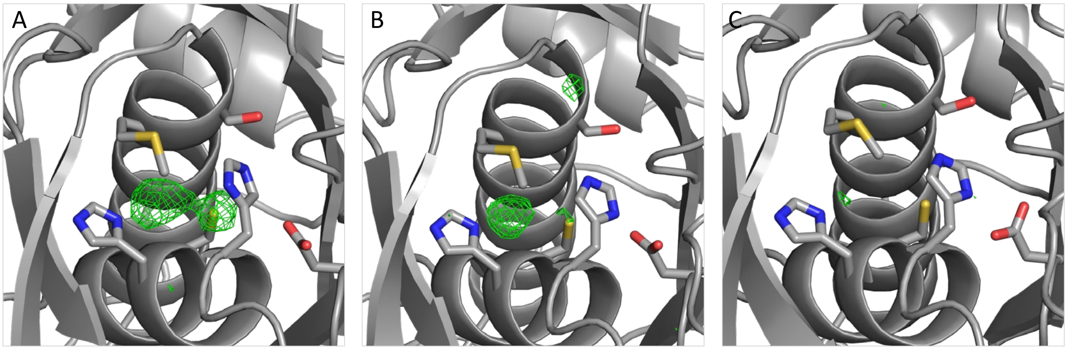

Supplementary Figure 2 | Positive electrostatic potential near the catalytic triad of proteinase K. The $m\mathrm{F_o}$-$\mathrm{F_c}$ maps are shown contoured at 3.5 σ above the mean. Positive electrostatic potential can be seen in the nanocrystal maps (A). The lamella maps also show some positive electrostatic potential for the data collected with a 20 µm condenser aperture (B), but not from the data collected with the 50 µm aperture.

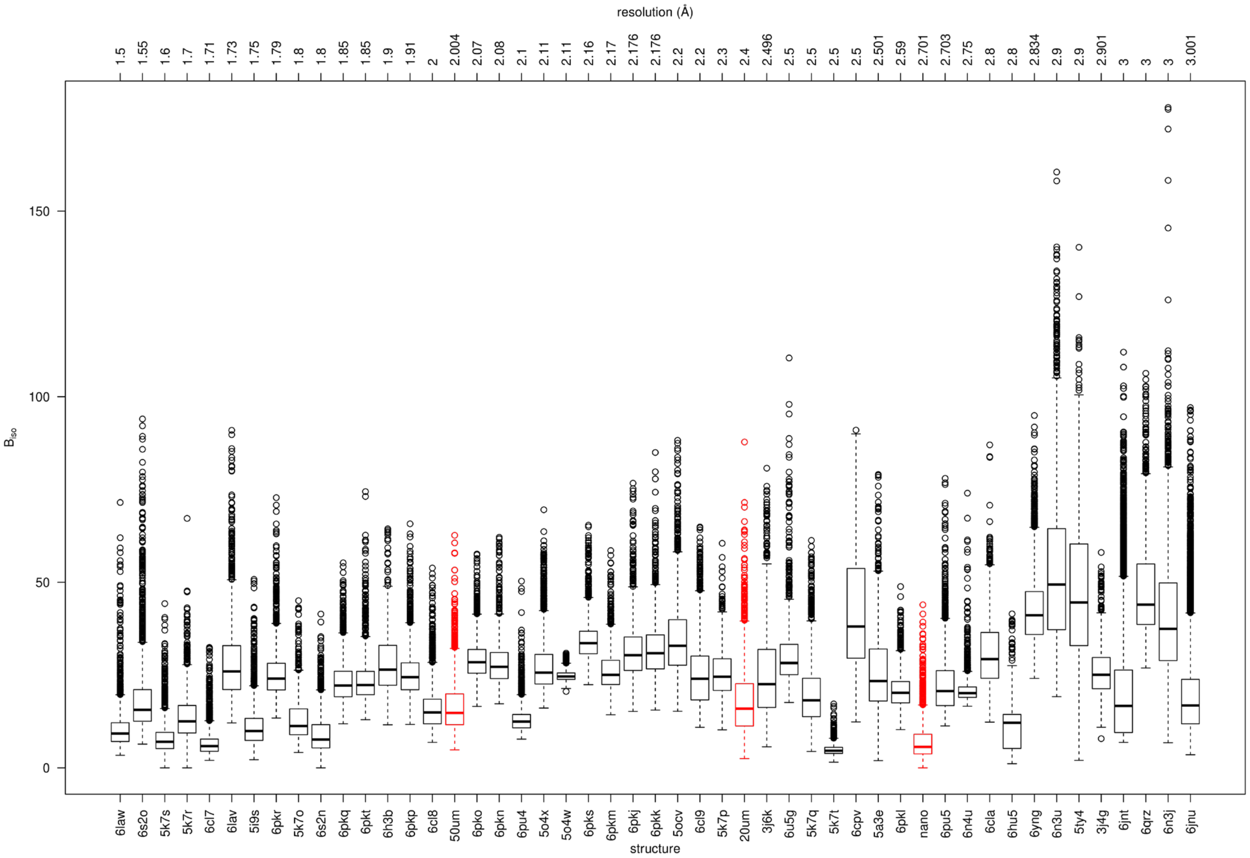

Supplementary Figure 3 | The distribution of isotropic B-factors are shown for all protein atoms from 46 protein structures determined by electron diffraction methods (excluding 2D crystals) and deposited in the PDB databank. The three additional structures shown in red are the structures from this study.

Supplementary Table 1. Comparison of experimental conditions between Martynowycz et al., 2019 and this study.

Experimental parameter	This paper	Martynowycz et al., 2019
Protein concentration	50 mg/mL	20 mg/mL
Protein solubilization buffer	25 mM Tris pH 7.5	50 mM Tris pH 8.5
Crystallization solution (precipitant)	20% (w/v) PEG 3350, 0.1 M ammonium chloride	1.25M ammonium sulfate, 50mM Tris-HCl pH 8.5
Crystallization method	Batch	Vapour diffusion
Method for transfer of crystals to grid	Aspiration by pipette from suspension	Aspiration by pipette from drop
Crystal size	~ 10x10x12 um	~25-30 um (as assumed from figure 1)
Blotting humidity	90%	100%
Blotting time	6-7 s	10 s
Blotting machinery/method	Leica EM GP	FEI Vitrobot IV
FIB-milling Ga beam currents	300-30 pA	300-30 pA
FIB-milling Ga beam voltage	30 kV	30 kV
Approximate milling time	~1 hr	~10 min*
TEM accelerating voltage	200 kV	200 kV
TEM apertures	C2 50 um	Not stated specifically, referenced papers only state either a selected-area aperture is used or is not.
TEM detector	Ceta-D	Ceta-D

* The reported 10 min milling time is probably an error or missing additional information.

Supplementary Material

Jscript for continuous rotating electron diffraction data collection (by Felix de Haas, Thermo Fisher Scientific)

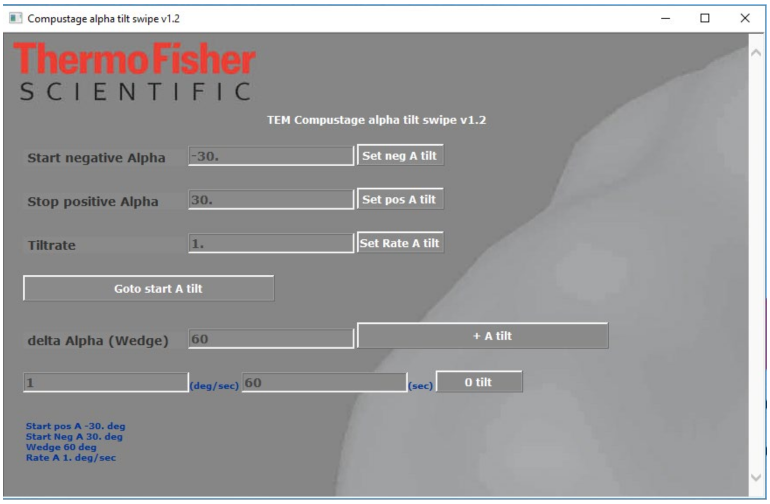

Supplement: Supplementary file 1 [file Data_Sheet_1.PDF]
